# The end of medical confidentiality? Patients, physicians and the state in history

**DOI:** 10.1136/medhum-2015-010773

**Published:** 2016-06-22

**Authors:** Philip Rieder, Micheline Louis-Courvoisier, Philippe Huber

**Affiliations:** 1 Institut Éthique Histoire et Humanités (IEH2), Université de Genève, Genève, Switzerland; 2 Hôpitaux Universitaires de Genève, Hôpital des Trois-Chêne, Genève, Switzerland

**Keywords:** History, Public health

## Abstract

Medical confidentiality has come under attack in the public sphere. In recent disasters both journalists and politicians have questioned medical confidentiality and claimed that in specific contexts physicians should be compelled to communicate data on their patients’ health. The murders of innocent individuals by a suicidal pilot and a Swiss convicted criminal have generated polemical debates on the topic. In this article, historical data on medical confidentiality is used to show that medical practices of secrecy were regularly attacked in the past, and that the nature of medical confidentiality evolved through time depending on physicians’ values and judgements. Our demonstration is based on three moments in history. First, at the end of the 16th century, lay authorities put pressure on physicians to disclose the names of patients suffering from syphilis. Second, in the 18th century, physicians faced constant demands for information about patients’ health from relatives and friends. Third, employers and insurance companies in the 20th century requested medical data on sick employees. In these three different situations, history reveals that the concept of medical confidentiality was plastic, modelled in the first instance to defend well-to-do patients, in the second instance it was adapted to accommodate the physician's social role and, finally, to defend universal values and public health. Medical secrecy was, and is today, a medical and societal norm that is shaped collectively. Any change in its definition and enforcement was and should be the result of negotiations with all social actors concerned.

## Introduction

Keeping secrets is both a social constraint and a professional imperative for a physician. It partakes in the definition of the professional identity of health professionals. And yet, why should physicians keep the darkest secrets of murderers, depressed pilots, rapists, unfaithful spouses, contagious patients and heads of state? At times, publishing such information appears to be a means of preventing public health issues, private and political disasters and seemingly, an unlimited list of crimes. The issue has been of late regularly broached in the public sphere. Media suggest that physicians should be compelled to inform relevant authorities of their patients’ health in order to prevent crimes and public disasters. Thus, taking up on present and past contestations of medical secrecy, occupational fields, status, gender or past offences are variables that have been or are being presented as requiring that the physician relax his practice of medical confidentiality.

In the eyes of journalists and politicians, medical data appear to carry a foreboding truth, demanding both attention and preventive measures. Three days after the horrific crash on 24th March of an airbus run by Luftansa's low-cost parent company, Goldenwings, German prosecutors announced the finding of a series of medical certificates excusing the co-pilot Adreas L from work. This information was immediately related to the announcement made the previous day by French prosecutors that data obtained from the black box suggested that the co-pilot had voluntarily crashed the plane.^1^ Less than a week after the crash, as from 30th March, the idea that airplane companies should automatically receive data on pilots’ health was voiced in the media.^2^ The ensuing polemic mirrors an ongoing debate in Switzerland as to whether physicians of dangerous prisoners should not automatically inform the judicial authorities of their patients’ (mental) health. The debate was triggered by the murder of Adeline M, a social worker, by Fabrice A, a convicted rapist and murderer. The murder was perpetrated on a therapeutic excursion during which the social worker accompanied Fabrice A. beyond the prison walls. Although the prosecution underway has revealed that a series of institutional dysfunctions clearly accounted for the fact that a notorious sex offender was let out in the sole company of a female therapist, the idea that medical confidentiality had no legitimacy in such situations surfaced in the media where it was presented as potentially endangering the lives of innocent members of the public. Judiciary and police authorities petitioned for means to compel health professionals working in prisons to disclose medical information about their patients.^3^

In view of these highly mediatised cases, it seems legitimate to question whether medical confidentiality should be guaranteed to the same extent to all patients. Modern-day preoccupations and obsessions with security issues appear to warrant a revision of medical deontology. Is medical confidentiality an obsolete concept? Different perspectives may be adopted in order to address such a question. In this article, we shall discuss historical inputs to the debate. What can history tell us about the genesis of medical confidentiality and the role played by healers in reacting to pressures coming from outside the profession? In the following pages, we shall show that while one may be tempted to consider past practices of keeping secrets as simple and obvious,^4^ historical data show that there was constantly a tension between social expectations and medical practices.

## Origins

The fact that the notion of medical confidentiality is almost as old as medicine itself is well established and confirmed by an often quoted section of the Hippocratic oath (IVth century B.C.):

What I may see or hear in the course of the treatment or even outside of the treatment in regard to the life of men, which on no account one must spread abroad, I will keep to myself, holding such things shameful to be spoken about.^5^

One of the most obvious characteristics of the oath is that the physician was to judge himself what exactly should not be communicated to third parties, that ‘which on no account one must spread abroad’. The fact that the oath itself was regularly quoted in texts since the 1st century B.C. has led many authors to believe that medical confidentiality was a constant medical norm. Historical research tells a more complex story. A short summary is necessary to understand later debates. To start with, the context of the formulation of the oath is not known precisely. Ludwig Edelstein has convincingly argued that the content of the oath itself suggests that it was written for physicians of the Pythagorean sect. This may explain why the content of the oath was not consistently applied by Greek physicians.^4^ ^5^ It was more popular in Roman times, not as a binding rule, but as a ‘medical reference’.^6^ All in all, little is known about actual practices of medical confidentiality in antiquity.

Understanding to what extent norms voiced in the oath were implemented in the following centuries is essential, and yet historical data on practices remain scant. The notion that the patient's secrets were to be kept by the physician was voiced by a series of medical authors in the Middle Ages and more regularly since the Renaissance.^4^ ^7^ The frame in which medical confidentiality was set varied from one place to another during the early modern period, and practices often depended on individual physician's ‘judgement’ of what he decided not to disclose. In 1598, the rule that no member should ‘reveal either the secrets or what he had seen, heard or understood’ of the patients’ secrets was added to the statutes of the Paris Faculty. Other French universities and some surgeons’ guilds adopted similar regulations.^7^

Institutional rules thus suggest that confidentiality was often expected of practitioners, sometimes compared with a priest's obligation to withhold the content of confessions. Court rulings confirm this view as judges condemned those who used medical information to slander their patients^6^ and yet it was admitted that health professionals should inform the authorities of infectious diseases and some municipal regulations stipulated that healers were to report wounds.^8^ In short, confidentiality was a rather vague quality expected of a physician. This did not change in the last decades of the 18th century when ideas about confidentiality were voiced in an emerging literature concerned with medical etiquette, deontology and ethics. John Gregory (1724–1773) and Thomas Percival (1740–1804), recognised today to be the founders of modern medical ethics, considered confidentiality as essential to the moral behaviour of physicians. And yet neither of them gave precise indications as to the nature of secrecy. For both Gregory and Percival, to judge precisely what medical information was to be kept secret remained subject to the appreciation of each individual physician.^9^ ^10^ The view of medical confidentiality they thus formalised was compatible with that of the Hippocratic oath and adaptable to an indefinite number of social situations.

Within a few decades, such a subjective practice of medical confidentiality was outdated by the Code pénal (1810): confidentiality became a legal norm as all practitioners who failed to withhold confidential information about their patients were liable to a hefty fine (500 francs) and up to 6  months prison (article 378).^11^ The legal obligation for physicians to keep their patients’ secrets (except when public health or state security was at stake) withdrew, in theory a least, part of the responsibility from the physician and construed a more precise frame for medical practice.

The story of medical confidentiality could thus be that of the displacement of core responsibility from the physician to the judge, the 19th century marking a transition from an informal flexible practice regulated by the physician's judgement to a legal and codified practice upheld by political actors and enforced by judges. And yet, normative and theoretical texts teach us little about the realities of confidentiality in medical practice. Opening the perspective to court cases, private documents and professional journals reveals that medical secrecy was manifold in the past. It was designed at times to protect the patient's interests, elsewhere to support the patient's health or family interests and occasionally to defend the physician's reputation. To test the importance of the changes introduced by the Code pénal and to understand how ‘ideal attitudes’ were effectively translated into practices, it is necessary to change perspective and to consider particular situations and problems induced by medical confidentiality.

The following analysis is focused on three crises set off by problems of confidentiality concerning the patient and professional healers, and employers, institutions and the state between the 17th and the 20th century. Each case reveals something about the type of behaviour that was challenged, the actors and their attitudes. As the interpretation of medical confidentiality varied from one moment to the next, from one location to another and in order to privilege a historical perspective, we have chosen a circumscribed locus, the Swiss French region of Switzerland, where primary sources such as state records, letters to physicians and egodocuments are available. Concentrating on particular affairs and the debates that they set off reveals social values, constraints, interests, expectations and representations, which underpinned the practices of confidentiality in the past. Beyond the accumulation of historical information, we shall argue that, considered together, these affairs tell us first that medical secrets were adapted to the values and expectations of the social actors concerned, second that the status quo was never immutable and practices of secret were ever vulnerable to attacks from social groups and institutions that came to consider it as an obstacle to their own ends, and lastly, that the capacity of physicians and groups of physicians to withstand such attacks and negotiate new compromises was instrumental in the constant redefinition of medical confidentiality. A long series of adaptations mark long-term trends in the history of medical confidentiality.

## Confidentiality challenged by lay authorities

The issues at stake were high for the physician between the Renaissance and the Revolution. His behaviour and his practice were inscribed in a complex web of values, including economical, intellectual, social, corporatist, political, legal and institutional components. The physician's right and capacity to adjust his attitude singlehandedly to the circumstances of both the patient's status and the prevailing social rules were oftentimes questioned by administrative and political instances. Analysing physicians’ responses to pressures exerted on medical confidentiality offers insights into the values and interests at stake.

At the time when the first cases of the pox (syphilis) appeared in the late 15th century, physicians were used to collaborate with local authorities about plague cases.^12^ ^13^ Tensions did surface between individual healers and authorities over publicising the names of the sick. The plague hit indiscriminately. Revealing patients’ names had implications for the families of declared cases and sometimes for the physician. A physician's resistance to comply stemmed either from his decision to protect his patient (to keep his custom or out of pity) or from his own desire to avoid quarantine.^13^ ^14^

The issue was slightly different regarding patients suffering from the pox. Such patients were stigmatised as having contracted the disease by their immoral behaviour, although a variety of other means of contracting the disease were also recognised.^15^ In the 16th and 17th centuries, the pox became both a public health issue and a source of conflict between physicians and local authorities. Patients with the pox strove to avoid recognition and did all they could to hide their condition. This triggered specific precautions among healers; some referred to such cases anonymously, referring to them as ‘secret’ or ‘shameful diseases’ even in their private account books.^16^ In Geneva, public charities took care of certain pox patients, namely those considered to be innocent victims (children and wives), but patients whose behaviour was considered amoral were banished.^14^

All over Europe, policies were enacted to report the sick, triggered by moral and public health concerns. The first known action taken in 1590 by Geneva's authorities against syphilitic patients as a group was triggered by an ecclesiastic court, Geneva's Consistory, which judged moral and religious behaviour.^17^ The court deferred the case to the secular authorities who had the power to enforce physical punishments. The project was to require that surgeons and physicians report the names of patients to the authorities so that they could be confined. Three healers refused, insisting on the possible consequences ‘if [the disease] affected some honorable person or their children, a fact one would not like to bring to light’.^18^ The argument was sufficient to calm the aldermen's enthusiasm.

The problem remained endemic and tensions recurred in 1621. A second group of healers refused to comply with official requests to give up the names of their patients. They justified their attitude. First, they declared that they had sworn not to reveal any disease ‘which should remain secret’ when graduating, rendering any disclosure impossible. The basis of their argumentation was their deontology and integrity. Second, they claimed that if they complied, the effect would be that the sick would no longer consult them, which would only make the situation worse. Here they addressed the politicians’ concern for public health. Third, they argued that the pox was not dangerous in Geneva, and thus the risk of contamination was not a major concern. Medical science was the final argument voiced to justify their refusal to comply. The authorities apparently gave in, although they did require that all healers disclose the names of poxed ‘ruffians and prostitutes’, without shocking anyone.^19^ ^20^

The pox set off negotiations between healers and authorities. Of interest here is the fact that arguments put forward in the late 16th and early 17th centuries are still voiced today in debates about mandatory declarations of contagious patients and the protection of patients’ data when social stigmatisation is a serious risk. Confrontations between state and healers concerning patients suffering from the pox suggest, not surprisingly in a tiered society, that the social status of the patient and possibly his or her weight as a paying client could trigger differentiated treatment. That vagrants, marginal social groups and the poor should be treated differently was not questioned. Social discrimination was, at that time, a problem neither for the physicians nor for the aldermen. More interesting for our purpose here is the fact that problems set off by the pox led, for the first time, physicians of Geneva to refuse to reveal a specific diagnosis. This may answer the tendency of patients to consult illegal specialised healers who promised effective, rapid and discreet treatments which regular practitioners had not hitherto guaranteed.^21^ Regular healers were thus induced to guarantee privacy if they wished to keep a foot in what was a very lucrative market. This is a first milestone in the genesis of a modern conception of medical confidentiality.

## Confidentiality challenged by patients

Medical secrecy did prevail in medical contexts, although not always in the sense expected today. Friends and family would often enjoin the physician not to disclose a bad prognosis to a patient for medical reasons: emotions were considered to be lethal to a fragile patient's health.^22^ Worry could also entice next of kin and spouses to secretly consult a physician for a loved one. Mme de Nettencourt did not inform her daughter before consulting the distant and yet famous Dr Tissot (1728–1797) about her case: “I am frightened that she would worry still more about my anxiety…” the mother explained to the physician, “she shall not know that I have the honor to write to you until I receive your answer”.^23^ Siblings, parents and friends were the objects of secret consultations. Anxiety was contagious in the patient's social circle of which the physician was more often than not a member. His role was to assist all the actors of a disease situation.

Pressure was often exerted by family and friends to gain information about the patient's health. The therapeutic relationship was more than a private encounter between two individuals. Lay individuals expected friends and acquaintances to share medical stories. Research in patient history has shown that it was rare for a patient not to share knowledge with friends and family; epistolary consultations demonstrate just how ‘normal’ the circulation of such information was.^24^ ^25^ At the age of 22, for instance, Horace-Bénédict de Saussure (1740–1799) was capable of consulting a physician about his mother's health, communicating detailed information about her dejections, her spittle, transpiration and menses during the previous 5 years.^24^ In fact, to retain information about one's health could be a problem. Here Saussure's own health story illustrates the point: he believed that he suffered from a hereditary disease from his mother's family and withheld information about his health. This strategy upset his close relations and one of them sent an anonymous letter to a foreign physician, Albrecht von Haller, about his health. The wording of the letter suggests that by taking medicines in secret, Saussure had upset members of his circle of friends and family. Sickness was then a collective event rather than an individual one, and sharing information was accepted and required.^22^ ^26^

Social pressure weighed heavily on healers to reveal information on the health of patients known to those with whom healers entered into social intercourse. The norm was for physicians and patients to be friends.^27^ Well-to-do patients and physicians often lived in the same communities and met in social venues, which could make patients ill at ease. They also often exchanged stories of individual health in social gatherings. Patients could not always trust local physicians to keep their health status secret when it carried heavy social stigma such as venereal diseases or hereditary conditions. Although today physicians in small isolated communities do share social venues with their patients, the physician is not expected to be a friend, and even if he is, patients expect him to keep secrets. In the past, the success of alternative, often shady healers, and the open admission by physicians that they were not consulted by patients suffering from venereal diseases, confirms the fact that confidentiality was not a readily available orthodox service.

Another issue that became a problem towards the end of the 18th century was the common practice of requiring medical information about a possible son or daughter-in-law. Marriage was an important and often definite step, conveying identity and meaning to the lives of the majority.^28–30^ Families developed strategies in order to avoid generating sick offspring. Parents of young adults gathered information about the health of intended spouses. The marquessa d'Agrain, for instance, was planning to marry one of her daughters to a man who appeared to be the ideal son-in-law: he was of high extraction and possessed both natural and acquired qualities, which had all been screened. And yet, the family hesitated because the young man had confessed that he suffered a slight atrophy of his legs. They had consulted a physician who had been incapable of determining the possible impact of such a condition. A letter addressed to Dr Tissot heralded one central question: what were the possible effects on the future children? “I beg you to answer as if you were Monsieur d'Agrain, or as if you were yourself to be married”.^31^ Although Tissot's answer is not known, a short comment he left on the letter is quite clear as to his opinion: “this unfortunate disease will slowly progress and undoubtedly, in the end, make a cripple of him”.^31^ The doctor did not hesitate to offer his prognosis on the health of an individual who was not his patient. Being asked questions about third parties was far from exceptional and physicians were routinely expected to answer such requests even when they concerned their own patients.

Louis Odier, a physician of Geneva, exposed the dilemma in his 1803 conference on medical discretion. If healers aimed to respect their oath only when the diagnosis was likely to stigmatise the patient, their silence could be interpreted to the disadvantage of the spouse to be. If they answered truthfully, they would be breaking their oath. Odier also pointed out that medical knowledge was not unequivocal and that mistakes occurred. An incorrect interpretation could affect a destiny for the wrong reasons. The term destiny is not too strong here: one can suspect that after having received Tissot's answer, Mlle d'Agrain's fiancé's chances of marrying his beloved were slim. Odier himself related the story of young lady who did not marry because of the opinion voiced by a physician, insisting on the devastating effect this had had on her life.^22^

## Confidentiality challenged by health systems

The ‘wedding dilemma’ and more generally, the impact of physicians’ chatter on individual destinies, were, at least theoretically, solved when breaching medical secrets became a legal offence in France after 1810. The idea was to defend both private interests and public order by guaranteeing that those needing treatment could get it without taking the risk of being betrayed. The recognised exceptions to medical confidentiality were contagious diseases and offences against state security.^4^ During the following two centuries, the medical profession gained both respectability and credibility: the increasing impact of public health on public policies, the growth of hospital medicine and, last but not least, spectacular innovations (anaesthesia, X-rays, antibiotics) brought it new visibility.^32^ ^33^ Members of the profession and its numerous local and federal societies came forward to counsel governments. This is the time when ethical and deontological discussions were increasingly integrated into the medical sciences, a transformation made visible by the first international congress of deontology in 1900.^34^ The growing impact of health professionals at different levels of everyday life led to new questions about the management of medical confidentiality.

Among these, issues related to the new insurance schemes for the working class arose. In Switzerland, the actors were national companies and administrations, insurance companies, physicians and patients. Towards the end of 1920, the question of medical certificates became controversial. Private and public employers complained about the lack of information transmitted by doctors on employees on sick leave. The debate developed around a routine practical medical issue: the form physicians had to fill for the insurance company for each and every sick employee. Each physician interpreted individually what was to be included, some offering detailed information, others refusing to give any idea of what the diagnosis was. The lack of consistence became an issue for administrators. The central Swiss medical committee(Comité médical suisse) tried to standardise practices, but failed, due to disagreements between insurance companies and physicians, but also among physicians themselves.^35^

In 1920, an interesting debate surfaced in response to the request for a standardisation of practices made by a company in Lausanne. A medical commission, chaired by Dr Pochon, was formed to discuss what a physician should or should not communicate to employers and insurances companies concerning an employee's health. The elements discussed were (1) the seriousness of the disease, (2) the contagiousness of the patient and (3) the prescriptions given so that employers could check patients’ compliance. The commission's stand was more than accommodating. In its report, it recommended that physicians reveal the seriousness of the sickness, but also that they mention a diagnosis if it was a ‘real’ disease, with the exception of venereal diseases or diagnosis that could be of damage to the patient's reputation. In so doing, the report accommodated employers who planned to adapt the benefits given to employees to the nature of the diagnosis: an employee suffering from a ‘real’ disease would be paid a full salary, whereas an employee suffering from less ‘real’ diseases (such as nervosity, anaemia or weakness) would be allocated half-pay only. The commission also recommended that physicians reveal cases of contagious diseases and the list of prescriptions given to each individual patient.^36^

These recommendations triggered violent reactions from colleagues. Dr Maillart (1860–1932), a well-known Geneva physician, contended that since doctors were to reveal diagnosis in some cases, to refrain from doing so because the disease was stigmatised (in cases of venereal diseases for instance) was damaging information in itself. Furthermore, even if a particular diagnosis was not damaging at the time, it could become so at a later date. Silence, he asserted, was always the best solution, a solution that never forced the physician to make a choice between his conscience and his duties.^37^ “I couldn't believe Dr Pochon's report”, wrote Dr Rychner, a second indignant colleague active in the canton of Vaud; Rychner had serious doubts about the category of ‘real’ diseases, “and I know that I am not the only one. But I feel the need to discuss it, because each day which ends without any protest being voiced will suggest to administrations, employers and insurance companies that the entire medical corporation agrees with the stunning conclusion: that the physician is to reveal his patient's diagnosis”.^38^ The debate revealed that confidentiality was treated differently in each Swiss canton. In Geneva, for instance, physicians were in favour of a strict observance of confidentiality and refused to communicate information about their patients to anyone but a doctor working for an insurance company.^37^ Such a solution was not possible in the canton of Vaud as the local medical society was opposed to doctors working under contract (it worried that physicians would become employees) and physicians had to communicate information directly to administrators. Unsurprisingly, Dr Pochon's report was not accepted by the local medical society.

The question of communicating (or not) data on patients to insurance companies reveals the stakes of medical confidentiality. First, it illustrates the difficulty to find cohesion within the medical profession, due to different corporative environments and the leeway individual physicians had, some being more inclined to be discreet than others. Second, it shows the importance of the moral component of medical secrecy. In the view of some actors, physicians and laymen, it was normal to control the behaviour of employees. The latters’ compliance could be checked. Sick workers were considered useless for the nation's workforce, and state officials and doctors were expected to rally with employers in order to compel them to return to work. Third, it is significant that the question of diagnosis included the distinction between ‘real’ and ‘less real’ diseases. Sufferers of ‘real’ diseases were socially and medically recognised to deserve a full reimbursement, and others were not considered to be worthy of it. Tiredness was assimilated to laziness, and nervosity and anaemia were considered to be chronic diseases and thus personal flaws. Dr Rychner was indignant and wondered wittingly if ridicule was not a real disease from which some of his colleagues may have been suffering.

## Conclusion

All in all, medical secrecy appears to be by nature plastic and forever adapted to the context in which it is inscribed. The analysis of selected moments in the history of medical secrecy presented above shows that despite a constant recognition of the importance of the notion of medical secrecy through time, in practice attitudes were adapted to both social, economic, political, medical values and historical contexts. In all three historical situations, patients were stigmatised and pressure was exerted in order to ensure that they were punished because of the nature of their diseases. Confidentiality was challenged because of the way society considered particular diagnosis, specific categories of people and particular behaviour at different moments in time. The destiny of individual patients was at stake: imprisonment for syphilitic patients, prolonged celibacy for Mlle d'Agrain's fiancé, economic vulnerability for employees. Each situation calls attention to the weight of responsibilities physicians had to shoulder as individuals and as a professional group.

The attitude of physicians and the values they stand by have evolved over time and yet, medical confidentiality has always called upon a physician's capacity to judge by him or herself. Stigmatising patients and even sharing information about patients’ health with political instances have not always been seen by all physicians as a problem depending on the social status of the patient and the nature of the request. It is difficult not to suggest that past physicians were more inclined to protect patients they knew and patients who were good custom. At the same time, the three situations are revealing in the historical changes they infer. In the 17th century, the information lay authorities wanted access to was conveyed by the sick themselves: venereal diseases were described by patients suffering from their genitalia and both actors knew how to identify the cause of the disease. The authorities expected physicians to reveal information they came by in their professional life. Again, in the case of patient pressure, particularly apparent in the 18th century, but possibly common in earlier centuries, at issue was information given by the sick person, although here the judgement of the physician concerning prognosis could be requested. A medical expertise of a different nature is expected of physicians in the 20th century as it is surmised that their capacity of diagnosis enabled them to distinguish between different categories of patients. The physician was here a tool for revealing the patients’ secrets, some of which may have been unknown to him or herself. The emphasis is placed on the individual physician's diagnosis and yet again the Hippocratic idea surfaces, and despite clear normative rules, the physician must judge him or herself.

The physician's capacity to judge is clearly excluded in the highly mediated cases of Andreas L. and Adeline M. mentioned in the introduction. In these debates, the basic assumption is that the physicians’ judgement as to whether or not he, or she, should request the permission to reveal to the relevant authorities sensitive data gained in a consultation is not a sufficient guarantee when the lives and the health of others are in jeopardy. At stake is, in short, a loss of confidence in the individual physician and extraordinary belief in the capacity of medicine to predict the future behaviour of highly unstable individuals. Would the relevant information enable any one and any one instance to take appropriate measures?

Historical data does little to suggest an answer to such a question. It does reveal that the professional and legal foundations of medical confidentiality have evolved. During the 19th and 20th centuries, a web of legal and deontological dispositions was construed to guide the modern practitioner. In modern-day debates, the possibility of ‘relaxing’ or ‘softening’ medical confidentiality and even the idea of compelling physicians to reveal information about their patients were put forward in the public sphere.^39^ In early 2014, two Swiss cantonal governments planned laws, which would constrain physicians to reveal data on their imprisoned patients. Professional values were voiced in opposition to these projects. Each and every argument put forward in both public and professional media contributes to qualify the nature of the confidentiality expected of a physician.^40^ ^41^ A certain conception of therapeutic relationships was defended, a relationship that should enable patients to voice their ailments and problems to a doctor, making it possible for the latter to offer the best possible diagnosis and the most adequate therapy. Without the promise of confidentiality, the patient could be tempted to withhold information and thus impede directly on his physician's capacity to heal^42^ and by undermining the notion of medical confidentiality itself, the legal setting could encourage political authorities to exert an administrative control abusively.^43^

Medical confidentiality was and is not only a medical matter, but a societal concern. Social groups and institutions such as political deciders, insurance companies, medical guilds and even groups of patients threaten medical secrecy. Medical confidentiality remains plastic and should not be adapted to answer the interests of any single interest group, but must be tailored only in response to changing values in society and via a consensus among all actors concerned.
